# Chemical stability study of vitamins thiamine, riboflavin, pyridoxine and ascorbic acid in parenteral nutrition for neonatal use

**DOI:** 10.1186/1475-2891-10-47

**Published:** 2011-05-14

**Authors:** Daniela O Ribeiro, Daniela C Pinto, Luis Mauricio TR Lima, Nádia M Volpato, Lúcio M Cabral, Valéria P de Sousa

**Affiliations:** 1 Faculty of Pharmacy, Federal University of Rio de Janeiro, Rio de Janeiro, RJ 21941-590, Brazil; 2 Faculty of Pharmacy, Federal University of Rio Grande do Sul, Porto Alegre, RS 90610-000, Brazil

## Abstract

**Background:**

The objective of this work was to study the vitamins B_1_, B_2_, B_6 _and C stability in a pediatric formulation containing high amounts of calcium in the presence of organic phosphate, amino acids, glucose, sodium chloride, magnesium sulfate, pediatric vitamins and trace elements under different conditions using developed and validated analytical methods.

**Methods:**

The study was carried out during 72 h with formulations packaged in recommended storage temperature (4°C) and 25°C, with and without photoprotection.

**Results:**

The results showed that the methodologies used for assessing the chemical stability of vitamins B_1_, B_2_, B_6 _and C in the formulation were selective, linear, precise and accurate. The vitamins could be considered stable in the formulation during the three days of study if stored at 4°C. When stored at 25°C vitamin C presented instability after 48 h.

**Conclusion:**

The pediatric formulation containing high amount of calcium in the presence of organic phosphate, amino acids, glucose, sodium chloride, magnesium sulphate, pediatric vitamins and trace elements packaged in bag-type trilaminate presented a shelf life of the 72 h, when maintained under refrigeration, between 2°C and 8°C. This shelf life was measured considering the vitamins studied. Further studies are needed including all the vitamins present in this formulation.

## Background

Vitamins are components of parenteral nutrition (PN) used for attending the daily requirements and supplying deficiencies in neonates and, in their majority, are instable [1-3]. The chemical degradation is the most usual cause of vitamin loss in the PN bag. Two reactions are described as the most common and important: oxidation of the ascorbic acid and the reduction of thiamine [4].

The ascorbic acid is the less stable vitamin added to the PN, being rapidly oxidized stimulated principally by high temperatures and catalysed by oligoelements as copper [5-8]. The first step of its degradation route, when converted to dehydroascorbic acid, is reversible and this compound also has biological activity [9]. Other stages of the route are irreversible and produce compounds without biological activity [10]. The degradation of the ascorbic acid is based directly on the amount of oxygen present in the medium, becoming relevant the packing material used [4-8,11-13]. Several amino acids also interfere in the stability of ascorbic acid not only due its cysteine (cooper ions chelating), but also by reducing its potential, which would decrease the effect of residual oxygen [5-8,14,15]. Another relevant factor related to vitamin C is the oxalic acid formation as degradation final product that has toxic potential and rapidly reacts with free calcium, inducing the precipitation of calcium oxalate [16].

The main route of degradation of the thiamine is caused by its reduction due to the presence of sodium metabisulfite, the antioxidant used in the amino acid crystalline solutions [4,7,11,16-18], concentrations of 1 mmlo/L, which are sufficient to promote degradation [6]. Riboflavin is one of the vitamins that present more sensibility to photodegradation [7,19]. It is irreversibly converted in luminoflavin, luminochromo and compounds of less importance in the presence of oxygen [3,4].

A PN formulation comprises large amounts of dissolved components with reactive properties leading to a high potential of incompatibility and instability. The physicochemical instabilities include: formation and precipitation of insoluble salts (i.e. calcium monohydrogen phosphate), degradation or complexation that can alter the bioavailability of the components [20]. These reactions are time-dependant, dependant on the concentration of reactants, pH-value, temperature, light exposure, the presence of catalytically active components and the container material [20].

Although the vitamins stability study in the PN is fundamental due to its importance in the efficacy and clinical security. The vitamins assay methods described in the literature are in majority for determination in biological matrix as blood, serum and maternal milk, or in pharmaceutical forms [1-3,21]. The selective quantification of the vitamins, in this complex matrix (PN) is an analytical challenge [9,22,23]. Ascorbic acid is the vitamin most studied in PN due to its labiality. Methods for vitamin C quantification are available in the literature by HPLC [8,11,12], spectrophotometry [6] and titration [24]. They can also be found in the literature method for analysis of vitamin B_1 _using HPLC [11,12,19], but the stability of vitamins B_2 _and B_6 _are less studied and there is no validated method available for quantification of these vitamins in PN. Despite the availability of some methods in the literature there are not official methods for analysis of vitamins in PN, as pharmacopeia. The establishment of an official method is hampered by the heterogeneity of the PN prescribed. Thus, the reliability of a method for analysing a compound in different formulations should be evaluated by the validation. Thus, in this work we developed and validated analytical methods for selectively assay of vitamins B_1_, B_2_, B_6 _and C in the PN formulation studied.

The objective of this study was to quantify vitamins B_1_, B_2_, B_6 _and C in PN formulations in order to investigate the degradation of these vitamins in a pediatric PN containing a high concentration of calcium (93 mg/100 mL) in the presence of organic phosphate (1.1 mEq/100 mL), oligoelements and amino acids, packaged in the trilaminate bag under different conditions.

## Methods

### Formulation preparation

The formulation studied was composed by: 3 g of Pediamino TAU amino acid (Baxter; São Paulo; Brazil), 8,64 g of glucose 50% (Fresenius; Campinas; Brazil), 4 mEq sodium chloride 20% (Darrow; Rio de Janeiro; Brazil), 4,5 mEq calcium gluconate 10% (Halex Istar; Goiânia; Brazil), 1.1 mmoL sodium glycerophosfate 21.6% (Fresenius), 0.25 mEq magnesium sulfate 0.8%, 0.1 mL Ped element (Darrow) and 10 mL MVI 12 opoplex for adult (ICN Farmacêutica; São Paulo; Brazil). The vitamins concentration in PN: 2 mg/mL of vitamin C, 8 μg/mL of folic acid, 1.2 μg/mL of biotin, 0,01 μg/mL of cianocobalamin, 0.03 mg/mL of pantotenic acid, 0.072 mg/mL of B_2_, 0,8 mg/mL of nicotinamin, 0.08 mg/mL of B_6_, 0.06 mg/mL of B_1_, 0.0364 mg/mL of vitamin A, 0.1 μg/mL of vitamin D, 0.2 mg/mL of vitamin E. The final volume of 100 mL was completed with water for injection and packaged in a trilaminate bag with capacity of 300 mL (Halex Istar).

### Reagents and chemical substance of reference

The reagents hydrogen peroxide PA, hydrochloric acid, sodium 1-hexanesulfonate, methanol and glacial acetic acid spectrophotometric grad were purchased from Merck (Darmstadt, Germany). The chemical substance of reference of thiamine hydrochloride (purity: 99.0%) and pyridoxine hydrochloride (purity: 99.9%) were purchased from Roche (Basel, Switzerland). The chemical substance of reference of ascorbic acid (purity: 99.0%) was purchased from Spectrum Chemical (New Brunswick, USA) and the riboflavin 5-fosfate sodium dehydrated (purity: 99.2%) from Merck. Other reagents were obtained from local suppliers.

### Formulation preparation

The PN admixture formulas for neonatal use were prepared aseptically in a 300-mL 3-layered bag composed by polyester, polypropylene and polyethylene (HalexIstar, Goiânia, Brazil) under a laminar-flow hood in accordance with the National Health Department specifications Nr. 272 (1988), designed for infusion through central access. The PN admixture formulas were prepared with market products from pharmaceutical industries and based on official regulations [25-29].

The choice of composition was based in the literature recommendations of preterms [26]. For micronutrients, today, there are controversies regarding the optimal dose of vitamins for preterms. A standard supplementation is often not enough to compensate this patient. It is necessary to use pharmacological doses that exceed the recommendations of the literature [30]. Because of this evidence, the concentrations of the vitamins were overestimated.

Each admixture was prepared separately in triplicate or quadruplicate (referred to as 3 or 4 lots) divided into two at different temperatures: 4°C ± 2°C, in refrigerator; and in 25°C ± 3°C, simulating room temperature. A different number of samples were tested from each of the lots depending on the technique applied. The experiments were performed on the day of the admixtures preparation and also 24 h, 48 h, and 72 h after preparation, time periods indicated as D0, D1, D2 and D3 respectively.

Samples were aseptically collected from each formulation at appropriate intervals using a plastic syringe for the physicochemical tests.

### pH evaluation

For the evaluation of pH, a Mettler Toledo potentiometer calibrated with pH 4 and pH 7 buffers was used. For each measurement, a 10 mL sample was collected and placed in an amber glass flask. The pH was measured by dipping the electrode directly into the solution, at room temperature. The pH determination was carried out each day in quadruplicate for each formulation in all the conditions studied.

### Development of analytical methodologies

The initial analytical conditions for the development of the chromatographic method for vitamins B_1 _and B_6 _were based in methods described in the literature for other purposes [1,2,21,23]. The wavelength of maximal absorption for each vitamin was determined using individual standard solutions of vitamins B_1 _and B_6 _(8 μg/mL) for obtaining an absorption spectrum in the UV region. Later, the retention time of each vitamin in the chromatographic system was determined. For the routine analysis, a single standard solution containing the B_1 _and B_6 _was prepared, using the mobile phase as diluent. The HPLC analysis was realized using diode array detection, by the area under the peak in the wavelength of maximal absorption, 250 and 295 nm for B_1 _and B_6_, respectively. For the mobile phase selection, different proportions of methanol: water were tested, 50:50, 40:50, 27:73, 25:75 and 20:80 (v/v). The mobile phase was kept at pH 3.0 using glacial acetic acid. A Shimadzu HPLC system was used equipped with a SPD-M10APDA detector. Data was acquired with Class-VP 6.1 software. The ideal chromatographic conditions obtained for the assay of the vitamins was Bondapack C_18 _column size 300 mm × 13 mm, 10 μm (Waters, Milford, USA); mobile phase consisting of a mixture of methanol: water (27:73; v/v) and 1.4% of sodium 1-hexanesulfonate for the ionic par formation; flux rate of 0.35 mL/min with detection at 250 and 295 nm and 30 μL of injection volume.

The fluorescence is a common phenomenon in aromatic molecules, as vitamin B_2 _[31]. Based in the method applied to the raw material vitamin B_2 _of the USP a selective and sensitive method to assay B_2 _in the PN [28] was developed. Initially, a preliminary study was realized to set the emission and excitation wavelength to detect the vitamin B_2 _in the PN. The emission range was between 400-700 nm, with intervals of 5.0 nm; with excitation at 360 nm, with 2.5 nm of interval; and scan velocity of 1000 nm/min in a Jasco fluorimeter.

Based on the reduction potential of vitamin C, the iodometric titration was applied to assay this vitamin in the PN based in the pharmacopeical methodology for raw material and tablets [23]. The titrant used was iodine 0.05 M SV. The sample was diluted in sulfuric acid 10% (w/v; 25 mL) and a solution of starch 1% (3 mL) and distilled water in sufficient quantity for completing 100 mL were used as indicators. The end point was determined by the formation of a blue coloration. Each mL of iodine 0.05 M VS (volumetric solution) corresponds to 8.806 mg of ascorbic acid [24].

### Validation of analysis methodologies for assaying vitamins B_1_, B_2_, B_6 _and C in the PN

The methodologies used for the assay of vitamins B_1_, B_2_, B_6 _and C in the PN were validated by the selectivity of the vitamins and their degradation products in the formulation, linearity, precision and accuracy in accordance with recommended procedures [8,32,33].

The specificity of the method for assay of the B_1 _and B_6 _in the presence of other formulation components of the PN was evaluated by the comparison of the chromatograms obtained from a PN containing the standard vitamins in study (B_1 _and B_6_) with PN without the vitamins (placebo). The purity determination of the chromatographic peaks was also used with the software of diode array detector. The specificity of the assay of vitamin B_2 _was determined by the comparison between the spectrum of PN with the standard B_2_, placebo and the PN, verifying that the peak observed in the spectrum is attributed to one component alone. For the vitamin C the titration of the placebo was carried out. The results obtained in the placebo were discounted later from the volume obtained with the PN containing the vitamin C.

The linearity was evaluated in three different days, by three vitamins standard with five concentration levels, in the ranges of 20-60 and 30-90 μg/mL (B_1 _and B_6_, respectively), 1-5 μg/mL (B_2_) and 50-150 mg (C). The linearity of the method was determined by linear regression analysis of the values obtained experimentally with the *softwar*e Excel^® ^(Microsoft, 2002).

The precision of the injection was evaluated in five concentration levels of vitamins B_1 _and B_6_: 20-60 μg/mL and 30-90 μg/mL, respectively, in three runs each. The standard deviation (SD) and the relative standard deviation (RSD) were calculated for each point, from the obtained area.

Precision was considered at two levels: repeatability and intermediate precision. It was determined by intra and inter-day assays. Stock solutions of these vitamins were prepared and aliquots were taken to prepare solutions at three levels of concentration: 80%, 100% and 120% of the sample work concentration. For B_1 _(30, 40 and 50 μg/mL); B_6 _(45, 60 and 75 μg/mL); B_2 _(3, 3.5 and 4.5 μg/mL); and C (80, 100 and 130 mg). The precision of the method was assessed by the SD and RSD of the values obtained experimentally in three days consecutively. The accuracy of the method was verified by determining the known recovery amount of standard vitamins in the spiked PN placebo.

### Stress stability study of vitamins B_1_, B_6_, and B_2 _in the PN

In order to test the selectivity of the developed methodologies for assaying the vitamins studied in PN in the presence of possible degradation products, an accelerated degradation of vitamins, under forced conditions [32] was performed. The assay was carried out separately for each vitamin using the respective analytical method developed. For this, standard solutions were prepared, one with 40 μg/mL of vitamin B_1 _and 60 μg/mL of B_6_, and another with 3.5 μg/mL of B_2_. Each vitamin standard solution was stressed using 3% and 10% of hydrogen peroxide for 24 h, at room temperature. After 0, 6 and 24 h of reaction the samples were withdrawn and analysed using the respective methods.

### Samples preparation

Samples from PN were withdrawn volumetrically to carry out the vitamins assay. For B_1 _and B_6 _were used aliquots of 7 mL and for B_2 _0.5 mL. Each sample was transferred to a volumetric vessel of 10 mL and the volume completed with mobile phase for B_1 _and B_6 _and with PN without vitamins for the B_2_.

Vitamin C assay was carried out on a volumetric aliquot of 50 mL. The sample of PN was transferred to an Erlenmeyer containing 25 mL of sulfuric acid 10%, 3 mL of starch solution 1% and distilled water in sufficient quantity for 100 mL. This mixture was immediately titrated with iodine volumetric solution 0.05 M.

### Statistic Treatment

The experimental results obtained were presented as mean and standard deviation (SD). Comparisons between the results were evaluated by unpaired Student's *t *test with a 95% of confidence limit. Values of p < 0.05 were considered statistically significantly.

## Results and Discussion

### Development and validation of analytical methods

**Figure 1:**
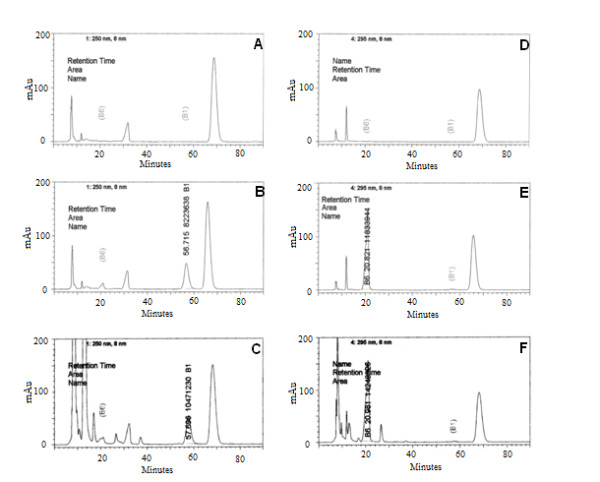
**Specificity of the vitamin B**_**1 **_**and B**_**6 **_**assay**. Chromatograms of PN placebo (A and D), spiked with the B_1 _and B_6 _(B and E) and PN containing the multivitamin (C and F) obtained by HPLC at wavelength of 250 and 295 nm, respectively, using column of reverse phase C_18 _and a mixture of methanol:water (27:73, v/v) and sodium hexano sulfonate 1,4%, pH 3.0 as mobile phase (n = 3).

The HPLC method was developed by assaying vitamins B_1 _and B_6 _in the same run. The selectivity of vitamins B_1 _and B_6 _assay was determined by comparing the chromatograms obtained from placebo, spiked PN with standard vitamins and samples of PN (Figure 1). The chromatograms show that despite the peaks presence of the other components, the vitamins B_1 _and B_6 _peaks were satisfactory separated from those others. The peak purity was of 0.9991 and 0.9999 for vitamin B_1 _and B_6_, respectively. The peak purity was calculated using the Shimadzu Class-VP 6.1 software.

**Figure 2:**
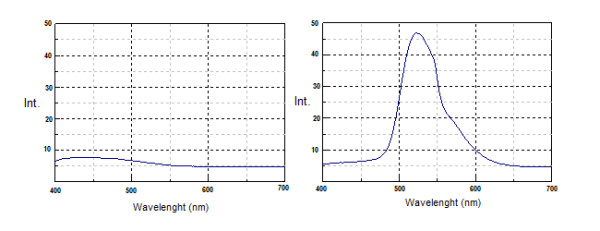
**Specificity of the vitamin B**_**2 **_**assay**. Fluorescence emission spectra of PN without (A) and with the standard vitamins B_2 _(B) with a range of emission wavelength between 400-700 nm and excitation at 360 nm.

The selectivity of the assay used for the determination of vitamin B_2 _in PN was confirmed by the comparison of fluorescence emission spectrums obtained from placebo and PN samples with B_2 _in the usual concentration (Figure 2). Three standard curves were also prepared, with five levels each, in the concentration range of 1 to 5 μg/mL, using solution of B_2 _in water, spiked placebo with B_2 _and PN sample. From these curves could be observed the spectrophotometric profile of the fluorescence light emission from vitamins B_2 _and the adequate linearity and selectivity of the method for this vitamin. This was evidenced due to the similarity of the vitamins B2 in water and spiked placebo with B_2 _curves, showing that with the established parameters the others PN components did not interfere in the assay. The curve obtained with the PN sample was above the other curves, but parallel due to the major concentration of vitamin B_2 _in this case. For the iodometric titration assay applied for the vitamin C in PN, the selectivity was evidenced by the low volume of the titrant, approximately 0.5 mL, necessary for placebo titration, compared with the volume used for titration of PN samples containing vitamin C, values between 5.0 and 16 mL.

The linearity relationship was evaluated across a range of each analytical procedure by three standard curves, realized in three different days, as presented in Table 1. The regression lines proved the adequate linearity of the methods (Table 1). The precision was determined by the RSD between the values obtained with the spiked samples with standard vitamins. In accordance with the Table 2, the precision of the methods were demonstrated adequately, since none of the values of RSD found for each level of concentration were less than 5% [28,33]. The system suitability test for HPLC was defined based on the results obtained in several chromatograms. The efficiency column determined by the analyte peak was >2000, the tailing factor <1.5 and the system precision was evaluated by six repeated assays of the same solution, the RSD was less than 2% [28].

**Table 1:** Regression analysis results of data for assaying vitamins B_1_, B_6_, B_2 _and C by HPLC, fluorescence and iodometric titration, respectively

Features	Vitamins			
	
	**B**_**1**_	**B**_**6**_	**B**_**2**_	C
Range	20-60 μg/mL	30-90 μg/mL	1-5 μg/mL	50-150 mg
Regression equation^a^	Y = 214695 x + 3168877	Y = 191100 x - 130681	Y = 57.9 x + 28.3	Y = 0.1x + 2.36
SE of intercept	2140.2	694.4	4.5	4.05
SE of slope	48166.4	3445.3	1.9	0.0
Correlation coefficient (*r*^*2*^)	0.9984 ± 1.45·10^-3^	0.9989 ± 1.54·10^-3^	0.9999 ± 0.00	0.9999 ± 5.0·10^-5^

^a^y = bx + a, where x is the concentration in μg/mL, except for vitamin C in mg, y is the peak area, for HPLC method, the intensity for fluorescence and volume for titration; a is the intercept and b is the slope. SE= standard error.

**Table 2:** Intermediate precision values obtained using the methods for vitamin B_1_, B_6_, B_2 _and C analyses in PN

		80%		100%		120%	
	
	Day	ID RSD (%)	IT RSD (%)	ID RSD (%)	IT RSD (%)	ID RSD (%)	IT RSD (%)
**B1**	1	1.78	1.40	1.89	2.00	0.48	0.88
	2	1.26		0.63		1.06	
**B6**	1	0.63	0.90	1.68	1.55	0.41	1.96
	2	1.01		0.95		0.98	
**B**_**2**_	1	0.40	1.18	1.09	1.59	1.08	1.61
	2	1.17		0.67		0.90	
**C**	1	0.71	1.46	0.57	1.33	0.43	0.56
	2	0.69		0.55		0.43	

ID RSD means intraday relative standard deviation and IT RSD interday relative standard deviation of 3 measurements average in each day. n = 2 lots.

The recovery data were obtained from the assay of the known amounts of standard vitamins spiked to the placebo. The obtained values were in a range of 98.6 to100.8% for all the developed methods, as demonstrated in Table 3. The mean values of the recovery data achieved show that there was no interference of the placebo and the accuracy of the methods was demonstrated.

**Table 3:** Experimental values obtained in the recovery test of vitamin B_1_, B_6_, B_2 _and C by HPLC, fluorescence and titration, respectively.

**[B**_**1**_**] (μg/mL)**	**Mass found (%) **± **RSD**	**[B**_**6**_**] (μg/mL)**	**Mass found (%) **± **RSD**	**[B**_**2**_**] (μg/mL)**	**Mass found **± **RSD (%)**	[C] (mg)	**Mass found (%) **± **RSD**
20	98.6 ± 3.05	30	99.2 ± 1.26	2	98.7 ± 1.35	50	98.7 ± 0.81
30	100.7 ± 2.21	45	100.6 ± 1.97	3	100.7 ± 1.22	80	99.2 ± 1.42
40	98.2 ± 3.82	60	99.8 ± 2.45	3	100.8 ± 1.13	100	98.9 ± 1.19
50	100.2 ± 0.90	75	99.8 ± 1.13	4	100.0 ± 1.27	130	100.0 ± 1.01
60	99.6 ± 0.69	90	99.9 ± 0.58	5	99.6 ± 0.89	150	99.5 ± 0.89

* Each value represents the mean of 3 analyses.

### Stress degradation study on vitamins B_1_, B_6 _and B_2 _in the PN

In addition to testing the specificity of the methods by the evaluation of PN with and without the standard vitamins, an accelerated study of the vitamins was carried out. This procedure aimed to ensure that the degradation products were not being quantified simultaneously with the studied vitamins. The stress degradation study for vitamin C was not necessary as the method applied is a pharmacopeical methodology.

**Figure 3:**
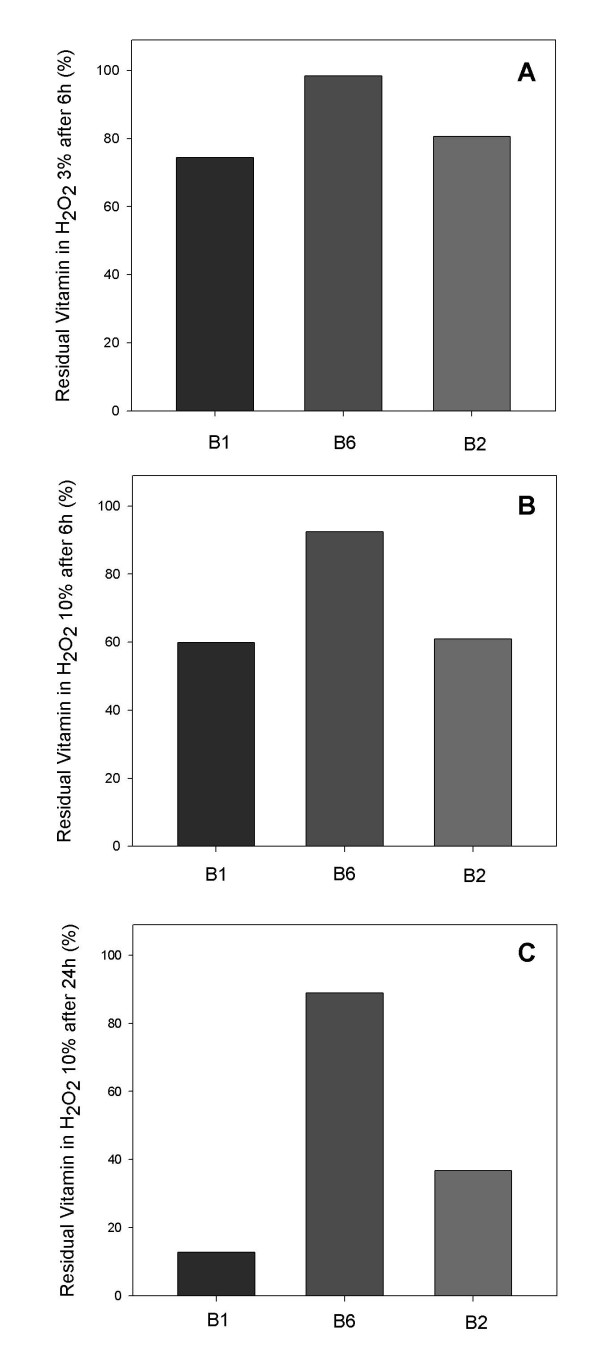
**Stress study of vitamins B**_**1**_**, B**_**2**_**, and B**_**6 **_**in PN**. Percentage remaining of vitamins after 6 h of oxidation using hydrogen peroxide 3% (A) and 10% (B) and after 24 h using hydrogen peroxide 10% (C).

In the panel A and B of Figure 3 the results of the chemical accelerated stability study of the vitamins B_1_, B_6 _and B_2 _after 6 h of oxidation using hydrogen peroxide 3% and 10% are demonstrated. The content remaining using hydrogen peroxide 3% and 10%, respectively, for B_1 _were: 74.7% and 59.8%; for B_6_: 98.4% and 92.4%; for B2: 80.6% and 60.9%. The panel C shows the results of the chemical accelerated stability study of the vitamins B_1_, B_6_, B_2 _after 24 h of oxidation using hydrogen peroxide 10%. The remaining content of B_1 _was of 12.7%; for B_6 _88.9%; and for B_2 _36.7%.

The results obtained by the accelerated degradation tests of the studied vitamins demonstrated the selectivity of the methods developed in the presence of degradation products in PN.

### Determination of the stability of vitamins B_1_, B_6_, B_2 _and C in PN

The choice of composition was based in the literature recommendations of preterms [26]. For micronutrients, today, there are controversies regarding the optimal dose of vitamins for preterms. A standard supplementation is often not enough to compensate this patient. It is necessary to use pharmacological doses that exceed the recommendations of the literature [30]. Due to this evidence, the concentration of the vitamins was overestimated.

**Figure 4:**
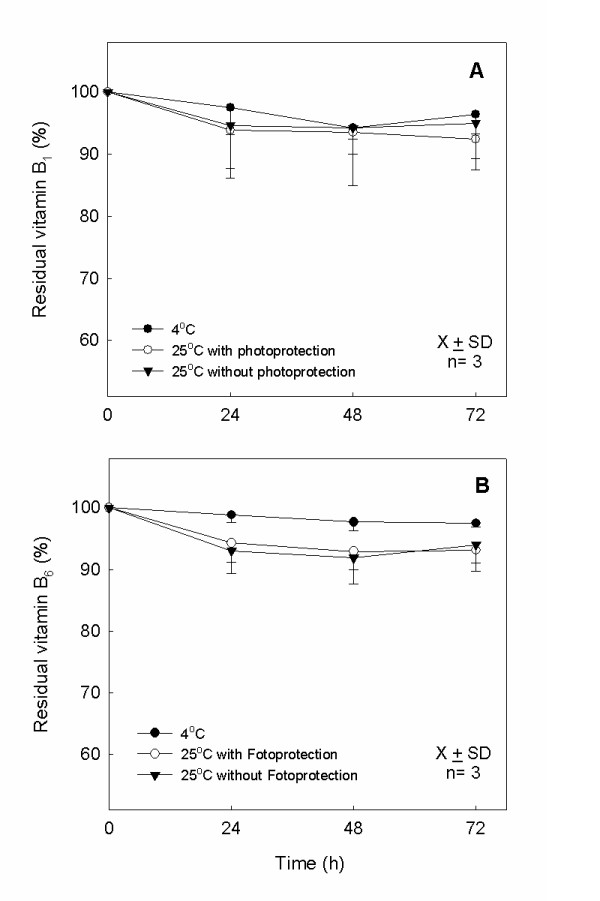
**Stability study of vitamin B**_**1 **_**and B**_**6**_. Residual content of B_1 _(A) and B_6 _(B) in PN stored at storage temperatures of 4°C and 25°C with and without photoprotection (n = 3 lots; P > 0.05, D3 compared with D0).

**Figure 5:**
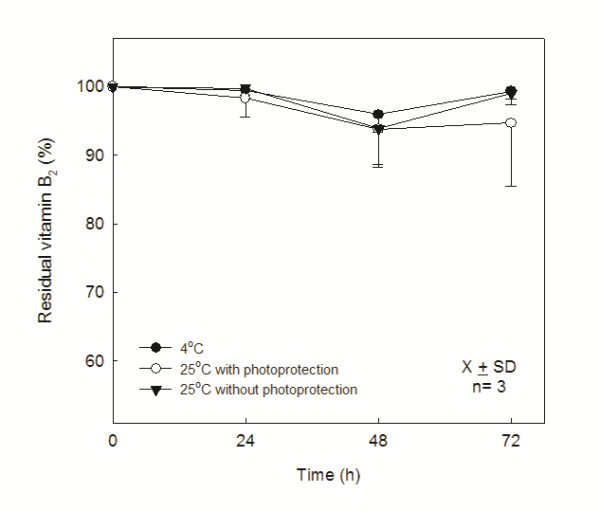
**Stability study of vitamin B**_**2**_. Residual content of vitamins B_2 _in PN stored at 4°C and 25°C with and without photoprotection (n = 3 lots; P > 0.05, D3 compared with D0)

Figure 4 shows the mean and the standard deviation of the reminiscent B_1 _and B_6 _quantity in the formulation until 72 h of storage. The residual quantities after stored at 4°C for B_1 _and B_6 _were: 96.4% ± 3.1% and 97.5% ± 1.0%; at 25°C with photoprotection: 92.4% ± 3.1% and 93.1% ± 6.0%; without photoprotection: 95% ± 7.6% and 94% ± 5.0%, respectively. The residual B_2 _quantity after 72 h of storage at 4°C was: 99.4% ± 1.1%; at 25°C with photoprotection: 94.7% ± 9.2%; and without photoprotection: 99.0% ± 1.6% (Figure 5). Statistically significative variations had not been observed (p > 0.05) for the vitamins during the days of study and under the studied temperatures.

**Figure 6:**
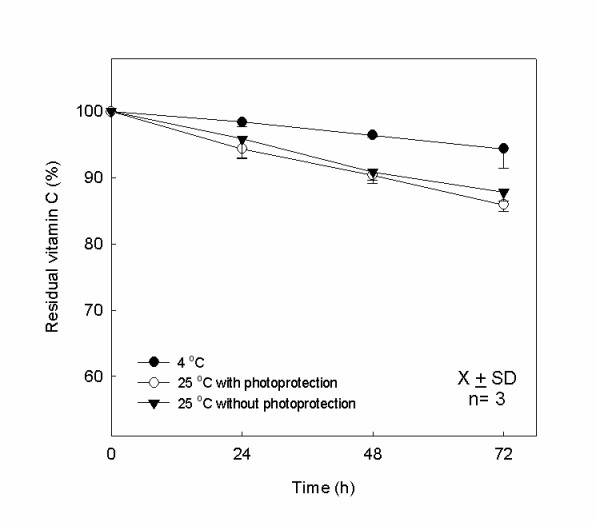
**Stability study of vitamin C**. Residual vitamin C in PN at storage temperature 4°C and 25°C with and without photoprotection (n = 3; lots; *P < 0.05, between the days D0 and D3 at 25°C with and without photoprotection).

The mean and the standard deviation of the remaining vitamin C percentage within 72 h of storage are presented in the Figure 6. The residual contents after storage at 4°C were: 94.4% ± 2.9%; at 25°C with photoprotection: 85.9% ± 1.1%; and without photoprotection: 87.8% ± 1.3%, showing statistically significant chemical alteration (p < 0.05) of vitamins C in the temperature of 25°C with and without photoprotection during the study.

According to ICH, the shelf life of a formulation can be estimated as the period time that the concentrations of the components are not reduced more than 10% [34]. No significant stability alteration (p > 0.05) was observed in the study of vitamins B_1_, B_2 _and B_6 _during 72 h in all studied conditions, i.e., less than 10% of content loss, as demonstrated in the Figures 4 and 5. However, the vitamin C presented significant alteration (p < 0.05), around 15% of loss after 72 h, when stored at 25°C independent of photoprotection, as demonstrated in Figure 6. These results were expected, because there was not incidence of direct natural light in the PN studied, only indirect natural light or artificial light. The observed degradation of vitamin C in the formulation could be due to other interfering factors such as the presence of oxygen, the storage temperature and the presence of oligoelements. It is important to reinforce that the important parameter for the variation of the vitamin C loss was the storage temperature, as observed in others studies [6,11] since the PN with and without photoprotection, when kept at 25°C, showed assay values very close and in the storage temperature indicated by the literature, between 2°C and 8°C, no significant alteration in the assay was observed. The loss of vitamin C observed was similar to other studies containing trace elements and vitamins in PN stocked in trilaminar bag [5,7,8]. Moreover, the relative stability of vitamin C is due to the bag used in this study. The trilaminate bag is 100 times less permeable to the oxygen than the EVA material, so the oxidation reaction occurs in minor intensity [23]. However, it has been reported the importance of removing the residual oxygen inside the bag after its preparation [6]. The mean and standard deviation of pH values for PN admixtures during the three days of study, at the two different temperatures selected were measured. In all cases the pH remained around 5.5 throughout the study period and showed no significant differences between the values of D3 and D0 in each condition tested.

It is established that the pH variation and the presence of reduction agents for vitamin B1 are factors that contribute to the loss of stability [16]. In the pH range between 5 and 6, the amino acids composition and reduction agents studied did not show significant alteration in the content of this vitamin (p > 0.05). It is described in the literature that concentrations of 1 mmol/L of sodium metabisulfite is sufficient to cause degradation of this vitamin and that when this reducing agent is diluted, it does not interfere in this degradation. This fact could be proven, since the PN studied, contained 0.078 mmol/L of sodium metabisulfite, below the quantity described as safe.

The presence of high concentration of calcium associated with organic phosphorus in the presence of oligoelements did not affect the stability of the analyzed vitamins, with the exception of the stability of vitamin C at 25°C, in which there was a loss of 15% in 72 hours of study. This formulation was chosen due its similarity to *in uterus *conditions, which favours greater bone mineralization [35], but with potential risk due to the low final volume and possible interactions leading to loss of vitamins, that, in this formulation was overestimated [4,36-38]. Some works have suggested a possible chemical instability due interaction between vitamins and oligoelements mixed in the same bag. A previous study demonstrated the compatibility and physicochemical stability of this formulation, but the stability of the vitamins in this formulation remained to be investigated at that time [38]. The lipid emulsion (LE) has a role of photoprotector in the PN, thus its absence favours the degradation of photo sensible vitamins as B_2 _and C [7,8,13,16]. So, possibly, the stability of these vitamins will be increased in formulations containing LE.

In spite of PN being an extemporaneous product, knowing the stability characteristics of this formulation makes possible to establish a quarantine period in order to realize effective microbiological control to promote major security in its administration. However, in order to obtain a complete outlook of the chemical stability of the vitamins in this formulation, further studies are needed including all the vitamins present in this formulation, since the PN contains vitamins beyond those studied here.

## Conclusions

The results obtained show that the methodologies used for assessing the chemical stability of vitamins B_1_, B_2_, B_6 _and C in the formulation were selective, linear, precise and accurate. The vitamins B1, B2 and B6 added in a PN containing high concentration of calcium associated with organic phosphorus in the presence of oligoelements could be considered stable during the three days of study, when stored between 4°C and 25°C. The vitamin C presented stability for 48 h at 25°C, with or without photoprotection. Considering the vitamins studied, the shelf life of the formulation studied is 72 h, if maintained under refrigeration, between 2°C and 8°C. Further studies are needed including all the vitamins present in this formulation.

## Competing interests

The authors declare that they have no competing interests.

## Authors' contributions

DOR participated in the design of the study and performed the experiments. DCP carried out the HPLC experiments. LMTRL performed the fluorescence measurements. NMV participated in the design of the study. LMC contributed to the data interpretation and review of the manuscript. VPS conceived of the study and had primary responsibility for writing the manuscript. All authors read and approved the final manuscript.

## References

[B1] Ivanovic D Popovie A Radulovie D Medenica M Reversed-phase ion-par HPLC determination of some water-soluble vitamins in pharmaceuticals J Pharm Biomed Anal 1999 18 999 1004 10.1016/S0731-7085(98)00109-5 9925335

[B2] Holler U Brodhag C Knobel A Hofmann P Spitzer V Automated determination of selected water-soluble vitamins in tablets using a bench-top robotic system coupled to reversed-phase (RP-18) HPLC with UV detection J Pharm Biomed Anal 2003 31 151 8 10.1016/S0731-7085(02)00574-5 12560059

[B3] Aniceto C Canaes LS Fatibello OF Cavalheiro CCS Determinação espectrofotométrica de vitamins B2 (riboflavina) em formulações farmacêuticas empregando sistema de análises por injeção em fluxo Quim Nova 2000 23 5 637 40 10.1590/S0100-40422000000500013

[B4] Allwood MC Kearney JC Compatibility and stability of additives in parenteral nutrition admixtures J Pediatric Child Health 2003 39 8 613 7 10.1046/j.1440-1754.2003.00246.x 9760591

[B5] Bara B Serna J García L López C Arroyo C Cardona D Bonal J Estudio de la estabilidad de la vitamina C en presencia de cobre, en mezclas de nutrición parenteral en bolsas multicapa Nutr Hosp 1995 10 41

[B6] Proot P De Pourco L Raymakers AA Stability of ascorbic acid in a standard total parenteral nutrition mixture Clin Nutr 1994 12 273 4 10.1016/0261-5614(94)90049-3 16843399

[B7] Martens HJM Stability of vitamins in TPN Clinical Nutrition 1988 7 74

[B8] Kearney MC Allwood MC Martin H Neal T Hardy G The influence of aminoacid source on the stability of ascorbic acid in TPN mixtures Nutrition 1998 14 173 8 10.1016/S0899-9007(97)00430-9 9530644

[B9] Allwood MC Factors Affecting the stability of vitamin C in total parenteral nutrition solutions J Clin Hosp Pharm 1984 9 75 85 6430966 10.1111/j.1365-2710.1984.tb01063.x

[B10] Gibbons E Allwood MC Neal T Hardy G Degradation of dehydroascorbic acid in parenteral nutrition mixtures J Pharm Biomed Anal 2001 25 605 11 10.1016/S0731-7085(00)00589-6 11377041

[B11] Dupertuis YM Morch A Fathi M Sierro C Genton L Kyle UG Pichard C Physical characteristics of total parenteral nutrition bags significantly affect the stability of vitamins C and B1: a controlled prospective study JPEN 2002 26 310 6 10.1177/0148607102026005310 12216712

[B12] Dupertuis YM Ramseyer S Fathi M Richard C Assesment of ascorbic acid stability in different multilayered parenteral nutrition bags: critical influence of the bag wall material JPEN 2005 29 125 30 10.1177/0148607105029002125 15772391

[B13] Allwood MC Brown PW Ghedini C Hardy G The stability of ascorbic acid in TPN mixtures stored in a multilayered bag Clin Nutr 1992 11 284 8 10.1016/0261-5614(92)90005-B 16840010

[B14] Melanie CJ Kearney JC Michael C Allwood MC Helen M Trevor N Gil H The influence of amino acid source on the stability of ascorbic acid in TPN mixtures Nutrition 1998 14 173 8 10.1016/S0899-9007(97)00430-9 9530644

[B15] Such AD C Sánchez CG Gomis PM Herreros AT Estabilidad de vitaminas en nutrición parenteral Nutr Hosp 2009 24 1 1 9 19266106

[B16] Allwood MC Melanie CJ Compatibility and Stability of Additives in Parenteral Nutrition admixtures Nutrition 1998 14 697 706 10.1016/S0899-9007(98)00063-X 9760591

[B17] Montero CG Vílchez T Cantabrana F Atienza M Stability of thiamine in parenteral nutrition fluids J Clin Nutr Gastroenterology 1990 5 89 93 2127544

[B18] Kearney MCJ Allwood MC Neale T Hardy G The stability of thiamine in total parenteral nutrition mixtures stored in EVA and in multi-layered bags Clin Nutr 1995 14 295 301 10.1016/S0261-5614(95)80067-0 16843946

[B19] Montero CG Vílchez T Atienza M Estabilidad de riboflavina y piridoxina en nutrición parenteral SEFH 1990 1 25 8 2127544

[B20] Mühlebach S Franken C Stanga Z Practical handling of AIO admixtures - Guidelines on Parenteral Nutrition, Chapter 10 GMS Germ Med Sci 2009 7 1612 3174 10.3205/000077 PMC2795373 20049073

[B21] Markopoulou CK Kagkadis KA Koundourellis JE An optimized method for the simultaneous determination of vitamins B1, B6, B12 in multivitamin tablets by high performance liquid chromatography J Pharm Biomed Anal 2002 30 1403 10 10.1016/S0731-7085(02)00456-9 12408932

[B22] Sforzini A Bersani G Stancari A Grossi G Bonoli GC Ceschel Analysis of all-in-one parenteral nutrition admixtures by liquid chromatograph and laser diffraction: study of stability J Pharm Biomed Anal 2001 24 1099 1109 10.1016/S0731-7085(00)00564-1 11248506

[B23] Alwood MC Martin HJ The photodegradation of vitamins A and E in parenteral nutrition mixtures during infusion Clin Nutr 2000 19 339 42 10.1054/clnu.2000.0109 11031072

[B24] US Pharmacopeia 31 acid ascorbic monograph 2008 United States Pharmacopeial Convention, Rockville 1445

[B25] Food and Drug Administration Safety Alert: Hazards of precipitation association with parenteral nutrition Department of Health and Human Services. Am J Hosp Pharm 1994 51 427 7

[B26] ASPEN Safe practices for parenteral nutrition JPEN 2004 28 173 204 10.1177/0148607104028006s39 15568296

[B27] BRASIL BRASIL Regulamento técnico para a terapia de nutrição parenteral Secretaria Nacional de Vigilância Sanitária do Ministério da Saúde 1998 http://www.anvisa.gov.br/legis/resol/2003/re/899_03re.htm Portaria nº272

[B28] US Pharmacopeia 31 General chapters, Validation of compendial procedures Section 1225, United States Pharmacopeial Convention, Rockville 2008 683 7

[B29] US Pharmacopeia 31 General chapters, Pharmaceutical Compounding--Sterile Preparations Section 797 2008 United States Pharmacopeial Convention, Rockville 319 336

[B30] García de Lorenzo A Álvarez J Bermejo T Gomis y P Piñeiro G Micronutrientes en nutrición parenteral Nutr Hosp 2009 24 2 152 155 19593484

[B31] Lacowicz JR Principles of Fluorescence Spectroscopy 1999 New York: Plenum Press

[B32] International Conference on Harmonization ICH Q2B: Validation of Analytical Procedures: Methodology Federal Register 1997 62 27463 7 http://private.ich.org/LOB/media/MEDIA417.pdf

[B33] BRASIL Guia para validação de métodos analíticos e bioanalíticos Diário Oficial da União 2003 http://www.anvisa.gov.br/legis/resol/2003/re/899_03re.htm21584968

[B34] International Conference on Harmonization ICH Q1A: Stability testing of new Drug Substances and Products Federal Register 2003 68 65717 18 http://private.ich.org/LOB/media/MEDIA419.pdf14631936

[B35] Devlieger H Meyers Y Willems L De Zegher F Van Lierde S Proesmans W Eggermont E Calcium and phosphorus retention in the preterm infant during total parenteral nutrition. A comparative randomized study between organic and inorganic phosphate as a source of phosphorus Clin Nutr 1993 12 277 81 10.1016/0261-5614(93)90046-7 16843326

[B36] Rigo J Senterre J Nutritional needs of premature infants: current issues J Pediatr 2006 149 S80 S88 10.1016/j.jpeds.2006.06.057

[B37] Lee MD Yoon JE Kim SI Kim IC Stability of total nutrient admixtures in reference to ambient temperatures Nutrition 2003 19 886 890 10.1016/S0899-9007(03)00173-4 14559326

[B38] Ribeiro DO Lobo BW Volpato NM da Veiga VF Cabral LM Sousa VP Influence of the calcium concentration in the presence of organic phosphorus on the physicochemical compatibility and stability of all-in-one admixtures for neonatal use Nutr J 2009 8 51 60 10.1186/1475-2891-8-51 19857269 PMC2772853

